# An Effective Resistive-Type Alcohol Vapor Sensor Using One-Step Facile Nanoporous Anodic Alumina

**DOI:** 10.3390/mi14071330

**Published:** 2023-06-29

**Authors:** Chen-Kuei Chung, Chin-An Ku

**Affiliations:** Department of Mechanical Engineering, National Cheng Kung University, Tainan 701, Taiwan

**Keywords:** alcohol vapor, resistive sensor, nanoporous alumina, AAO, anodization

## Abstract

With the increases in work environment regulations restricting alcohol to 1000 ppm, and in drink-driving laws, testing for alcohol with a simple method is a crucial issue. Conventional alcohol sensors based on sulfide, metal oxide, boron nitride or graphene oxide have a detection limit in the range of 50–1000 ppm but have disadvantages of complicated manufacture and longer processing times. A recent portable alcohol meter based on semiconductor material using conductivity or chemistry measurements still has the problem of a complex and lengthy manufacturing process. In this paper, a simple and effective resistive-type alcohol vapor sensor using one-step anodic aluminum oxide (AAO) is proposed. The nanoporous AAO was produced in one-step by anodizing low-purity AA1050 at room temperature of 25 °C, which overcame the traditional high-cost and lengthy process at low temperature of anodization and etching from high-purity aluminum. The highly specific surface area of AAO has benefits for good sensing performance, especially as a humidity or alcohol vapor sensor. With the resistance measurement method, alcohol vapor concentration of 0, 100, 300, 500, 700 and 1000 ppm correspond to mean resistances of 8524 Ω, 8672 Ω, 9121 Ω, 9568 Ω, 10,243 Ω, and 11,045 Ω, respectively, in a linear relationship. Compared with other materials for detecting alcohol vapor, the AAO resistive sensor has advantages of fast and simple manufacturing with good detection limits for practical applications. The resistive-type alcohol vapor-sensing mechanism is described with respect to the resistivity of the test substance and the pore morphology of AAO. In a human breath test, the AAO sensor can quickly distinguish whether the subject is drinking, with normal breath response of −30% to −40% and −20% to −30% response after drinking 50 mL of wine of 25% alcohol.

## 1. Introduction

In recent years, with stricter work environment regulations and drunk-driving laws, the testing of alcohol has become a major focus. Generally, chromatography is used for detecting organic substances, but it needs to be sampled and packaged. Processes such as transportation and analysis are time-consuming, so fast and simple methods to detect the concentration of organic gas has rapidly developed. Conventional alcohol sensors are based on 2D materials such as sulfide [1,2,3,4], metal oxide [5,6,7,8,9,10], boron nitride [11] or graphene oxide [12,13,14,15], and the detection limit is about 50 to 1000 ppm. However, these methods have disadvantages such as complicated solution synthesis steps [1,2,3,4,5,6,7,8,9,10,11,12,13,14] and/or long processing times of 12–72 h [1,2,3,4,5,6,7,8,9,10,11,12,13,14] with lower sensor response [6,13]. Furthermore, etching or toxic solutions are often used in the above processes, which are harmful to the environment. A recently-developed portable alcohol meter uses semiconductor materials for conductivity or chemistry measurements, but the problem of complex and lengthy processing still remains. On the other hand, current research on alcohol sensing detection is based on using metal oxide with lower sensing limits [16,17,18,19,20]. In 2020, Zhang et al. used the hydrothermal method to synthesize WS_2_ and WO_3_ for alcohol detection [19]; however, the solution cost more and there was a longer process time of 43 h. In the following year, Annanouch et al. applied the lithography process to develop a SnO_2_-based MEMS chip to detect alcohol vapor [20]. The sensor response was significantly improved; however, the problem of a complicated and time-consuming process still remained. Furthermore, the saturation problem [1] of the sensor at higher concentrations is another limitation to sensing. The signal from the sensor should be obviously and precisely distinguished around a proper range defined from regulations or other requirements. Therefore, a suitable detection range of 50–100 ppm to the legal limit of 1000 ppm, suitability for human breath monitoring, and an economical sensor preparation process still need development.

Surface treatment technology is a commonly used method in industry or research to improve surface hardness and anti-corrosion. However, with the evolution of micro-nano technology, other application methods have received attention. Anodic aluminum oxide (AAO) is a well-known nano-structured template in industry and science research [21,22]. Compared with other polymer-based nano-templates, for instance, the nano-sphere of polycarbonate or polystyrene, the advantages of AAO includes the nano-scale pores, self-organization, controllable pore size, cost-effective methods, good biocompatibility, and thermal and chemical stability. In addition, aluminum is the most commonly used metal in production lines, and its processing has a high degree of industrial compatibility. Furthermore, the highly specific surface area [23] of AAO structure leads to good sensing performance, especially as a humidity sensor [23,24,25,26,27,28]. In 1987, Khanna and Nahar [29] published the first paper on measuring atmospheric humidity using AAO. Since then, research on capacitive-type AAO humidity sensors has continued. In 2000, Nahar [30] proposed a comprehensive adsorption mechanism to explain the signal change in humidity measurements, where the first layer of water molecules, or the chemisorbed layer, dominates the electrical signal increase at a relative humidity of <45%. In addition, the multilayer physical adsorption layers occur when humidity is >45%. In the past 20 years, many groups have explored the fabrication of AAO humidity sensors to enhance their performance and expand their application. In 2009, Kim et al. [31] proposed using patterned electrodes to achieve better sensing capabilities, and significantly improved the response time of AAO humidity sensors to 18 s. In 2010, He et al. [32] conducted research on the anion concentration and annealing of AAO pore walls, suggesting that anions contribute to enhance sensor response, while annealing increases the capacitance value. In 2014 and 2015, Chung et al. [27,33] optimized the sensors by varying oxalic acid concentration and applying a magnetic field, leading to significantly improved response. In recent years, some researchers have proposed integrating humidity sensors with common objects. For example, in 2015, Blade [34] embedded AAO humidity sensors on paper substrates for flexible devices. In 2020 to 2021, Chung et al. analyzed the relationship of AAO pore geometry and the sensor response. The response and response-recovery times are affected by the specific surface area and the thickness of AAO pore structure. In summary, the sensing mechanism of AAO has been extensively studied. Taking advantages of its simple and fast fabrication process, highly specific surface area and high stability, AAO shows great potential for developing alcohol sensors but there is still less research on AAO than on organic solvent or alcohol sensing in related methods. To our best knowledge, in 2020, Podgolin et al. [35] carried out the first research using high-purity AAO sensors for alcohol detection with a 2-step low-temperature anodization for about 48 h. However, it only works at a highly concentrated environment of 1~100 vol% alcohol in air, not at the required 1000 ppm. It is worth further developing an alcohol sensor with a proper detection limit with a simple fabrication process.

In this article, we propose a simple and cost-effective method to fabricate AAO resistive nano-sensors in 2 h for rapid detection of gas-phase alcohol vapor in the practical range 100~1000 ppm. The AAO was synthesized from low-purity aluminum alloy (~99.5%) at 25 °C, which was an improvement over the traditional high-cost and lengthy process at low temperatures of 0~10 °C for anodization and etching from high-cost and high-purity (99.99%–99.999%) aluminum. Compared with other materials to detect alcohol vapor, the AAO resistive sensor has the advantages of fewer fabrication steps and shorter process time, along with a practical detection range. The alcohol vapor-sensing mechanism is discussed with respect to the resistivity of the test substance and the pore morphology of AAO. The AAO alcohol sensor is also tested in a concentration-resistance experiment and a human breath test for potential applications.

## 2. Materials and Methods

**Figure 1:**
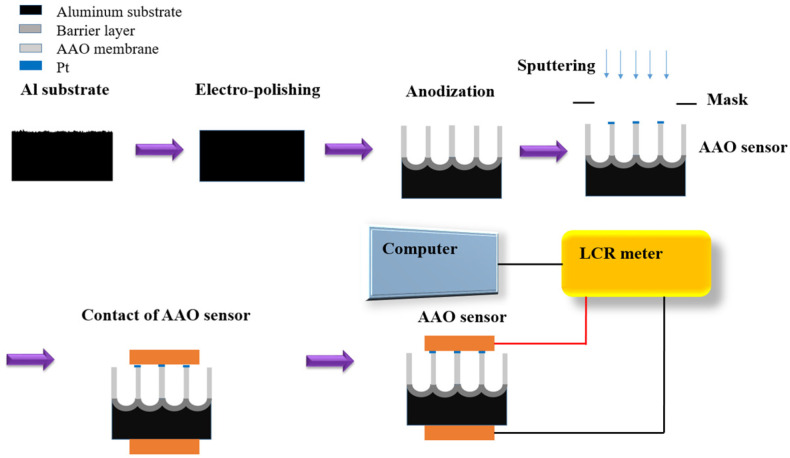
The experimental process flow for AAO alcohol sensor fabrication, including the electropolishing, anodization, Pt sputtering, the contact of the AAO sensor, and the measurement device setup.

A low-purity, 1050 aluminum alloy of 0.8 mm thickness was selected as the sensor substrate and cut into a working size of 2.5 × 2.5 cm^2^. The 1050 aluminum alloy was put into a pre-made specialized mold and underwent electrochemical polishing using a power supply. To remove the impurities and reduce the surface roughness, AA1050 substrate was electropolished using the mixed solution of perchloric acid and ethanol. From our previous work, the two-step polishing was conducted using a perchloric acid:ethanol = 1:1 (*v*/*v*) solution at 20 V for 1 min under 0 °C, and continued with perchloric acid:ethanol = 1:4 (*v*/*v*) for 5 min with the same parameters [36]. For the anodization process, an electrochemical potentiostat with a three-electrode system and a 4 L tank with cooler (DANG YNG 610) and cycling pump was constructed. Platinum mesh was selected for the counter electrode, the 1050 aluminum alloy in our self-made mold as the working electrode, and calomel was set as the reference electrode. AA1050 was anodized by the hybrid-pulse anodization (HPA) method for 2 h in 0.3 M oxalic acid at 20/−2, and 40/−2 V at 25 °C. The HPA process is a special rectangular waveform containing a relatively large positive voltage and a smaller negative voltage. In the period of negative voltage, the current measured was zero to suppress the heat accumulation and let the process conduct at room temperature without burning. Next, a metal deposition sputtering device with Pt target (JEOL JEC-3000FC) was used to sputter a Pt film for 150 s (about 8 nm thick) to form the top electrode. With the bottom electrode the AA1050 substrate itself, the metal-ceramic-metal (MDM) structure was formed for the electrical signal measurement. Figure 1 shows the process flow of the one-step anodization method for the AAO alcohol sensor. The morphology of the AAO nano-pore structure was observed using a high-resolution field scanning electron microscope (HRFESEM, JEOL JSM-7000F, Japan) to examine the pore integrity and the AAO thickness. The SEM image analysis was combined with the ImageJ software to calculate the pore diameter and the AAO thickness.

**Figure 2:**
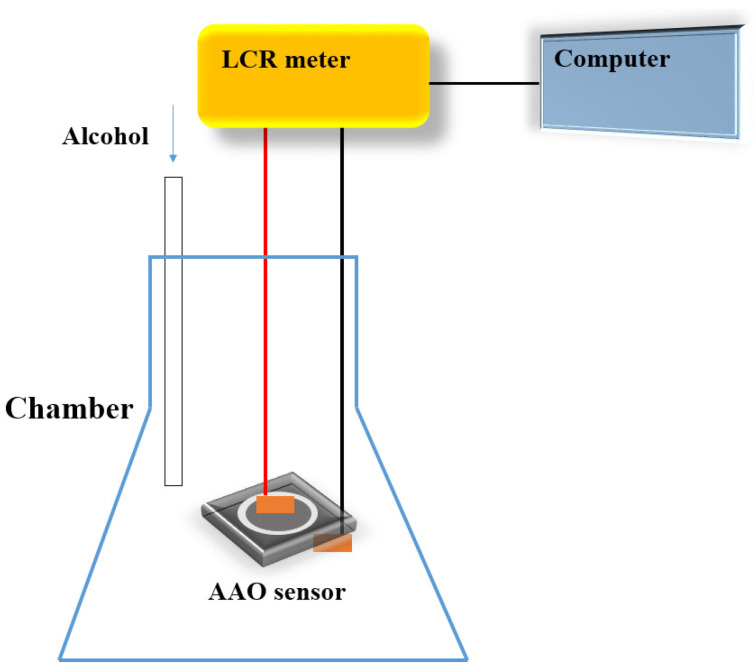
The experimental equipment configuration diagram for an AAO alcohol sensor measurement.

After the anodization process, the Pt was sputtered on the top of AAO with a mask to define the electrode area forming the MDM sensor structure, and the AAO sensor was placed into a chamber for examination. Although Pt may also deposit inside the pores of AAO, it has almost no effect on the performance of the sensor as it does not create an electrical current pathway. The measurement setup of the alcohol sensor preparation diagram is shown in Figure 2. The system includes a chamber, the AAO sensor, computer, and a LCR meter for resistive analysis under different conditions. The alcohol sensor measurement range is from 100 ppm to 1000 ppm. To further explain the sensing mechanism, the contact angle test for AAO sensor surface is conducted using a water or alcohol droplet of 6 uL. In addition, a practical application of human breath testing was carried out. During the human breath test, the subject first drank 50 mL of alcohol (25% alcohol concentration), and then exhaled at a fixed distance of 3 cm from the sensor.

## 3. Results and Discussion

**Figure 3:**
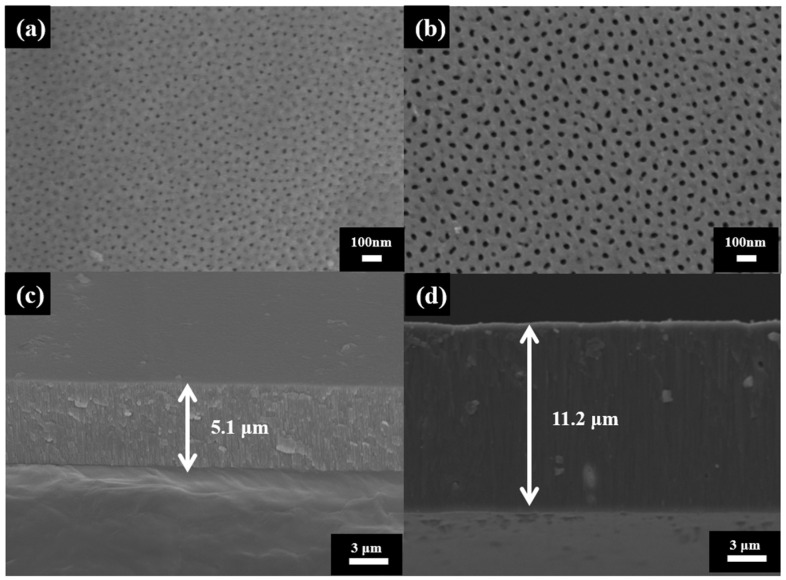
The SEM images of AAO (**a**,**b**) top views of AAO at 20 V and 40 V with pore diameter of 20.55 ± 5.32 nm and 31.76 ± 4.09 nm, respectively. (**c**,**d**) the cross-section views of AAO at 20 V and 40 V with thickness of 5.1 and 11.2 μm, respectively.

In the AAO experiment, we successfully achieved complete growth of porous AAO through one-step anodization at 20 and 40 V under room temperature. The SEM images of its nano-scale structure are shown in Figure 3. Figure 3a,b represent the SEM top-view images of AAO at 20 V and 40 V, analyzed by commercial ImageJ software to show an average pore diameter (Dp) of 20.55 ± 5.32 nm and 31.76 ± 4.09 nm, respectively. Figure 3c,d show the SEM cross-section views corresponding to Figure 3a,b, indicating a thickness of 5.1 and 11.2 μm, respectively. In the past few decades of research, the correlation between the parameters of AAO and the resulting pore structure has been extensively studied [37,38,39,40,41]. Factors such as temperature, voltage, acid type and concentration, and process time are the main influencing factors on the AAO nano-structure. Through validations by multiple research teams, precise control of anodization parameters has been shown to give consistent results. In our experiment, the AAO formation was measured three times, and all results were similar for Dp and thickness. Traditionally, the anodization process is often carried out in a two-step process at low temperatures. The lower temperature helps to suppress the generation of Joule heat during the reaction, and the two-step process allows for better-ordered AAO nano-structures due to the pre-positioning in the first step. However, the two-step low-temperature method leads to lower reaction efficiency and has a longer processing time. Therefore, in this experiment, we applied the HPA technique that takes advantage of the cooling effect achieved when the current is zero during the negative voltage phase, enabling the reaction to be conducted at room temperature. Compared to the growth rate of <3 μm/h in a traditional anodization process, the growth rate reached 5.6 μm/h in our AAO of 40 V, resulting in a more efficient preparation of AAO.

**Figure 4:**
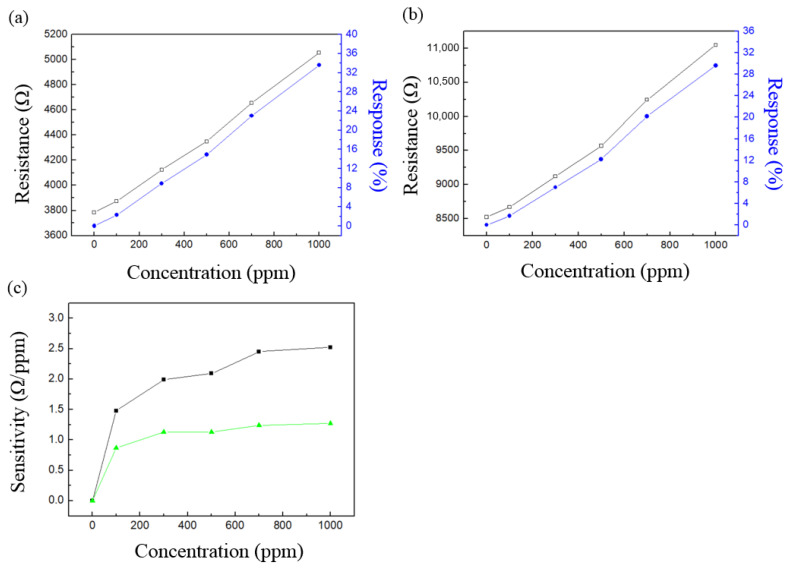
(**a**) Resistance values measured from AAO made at 20 V are 3785 ± 48 Ω, 3872 ± 52 Ω, 4123 ± 60 Ω, 4349 ± 60 Ω, 4655 ± 58 Ω, and 5056 ± 64 Ω, corresponding to 0 (air), 100, 300, 500, 700 and 1000 ppm alcohol vapor, respectively. The sensor responses are 2.3%, 8.9%, 14.9%, 23.0%, and 33.6%, respectively. (**b**) The resistances of AAO at 40 V for alcohol at concentrations of 0, 100, 300, 500, 700 and 1000 ppm are 8524 Ω, 8672 Ω, 9121 Ω, 9568 Ω, 10,243 Ω, and 11,045 Ω, respectively. The response values at concentrations of 100, 300, 500, 700 and 1000 ppm are 1.7%, 7%, 12.2%, 20.2% and 29.6%, respectively. (**c**) The sensor sensitivity in different ethanol concentrations from 40 V (black line, square) and 20 V (green line, triangle).

After the AAO is prepared, the Pt is sputtered to a thickness of 8 nm for defining the electrode area to form the metal-ceramic-metal structure of the alcohol sensor. The MDM nano-structure is advantageous for both resistance and capacitance sensing. With the adsorption of the target gas, the electrical properties of the sensor rapidly change. It is worth noting that the Pt deposited inside the pores of AAO has almost no effect on the sensor measurement as it does not create an electrical current pathway. In our related research, we found that only when the AAO thickness is less than 1 um is it affected by the sputtered platinum deposition. The AAO alcohol sensor measurement method and instrument setting are shown in Figure 2. For alcohol sensing, the sensor response R is an important performance indicator and is defined as the formula below:

(1) $$\mathrm{Response}\;=\frac{R-R_{0}}{R_{0}}$$

where R_0_ is the resistance value at the initial state in air, and R is the resistance value in a certain alcohol concentration. The response of the LCR meter to alcohol is measured at concentrations of 0, 100, 300, 500, 700 and 1000 ppm, and the measurement data are averaged after measuring the each concentration five times. In Figure 4a, the resistance values measured from AAO made at 20 V are 3785 ± 48 Ω, 3872 ± 52 Ω, 4123 ± 60 Ω, 4349 ± 60 Ω, 4655 ± 58 Ω, and 5056 ± 64 Ω, corresponding to the 0 to 1000 ppm scale. The sensor responses are 2.3%, 8.9%, 14.9%, 23.0%, and 33.6%, shown in Figure 4a as a black line. The resistance values measured from AAO at 40 V are 8524 ± 54 Ω, 8672 ± 71 Ω, 9121 ± 66 Ω, 9568 ± 74 Ω, 10,243 ± 77 Ω, and 11,045 ± 69 Ω, respectively, as shown in Figure 4b. The responses at alcohol concentrations of 100, 300, 500, 700 and 1000 ppm are 1.7%, 7%, 12.2%, 20.2% and 29.6%, respectively, also in Figure 4b. As the alcohol concentration increases, there is a linear growth trend in the resistance value. The responses was calculated from three different samples and showed similar results. However, the resistance values showed a little difference because of the relative humidity changes. When the relative humidity is higher, the initial resistance value of the AAO sensor becomes lower. However, there may still be a small amount of error in the resistance measurement. In the alcohol concentration experiment, the responses show more stable results. Therefore, we also used the response value in the subsequent breath analysis. In Figure 4c, the sensitivity is calculated based on the raw resistance data from Figure 4a,b. The formula for sensitivity is as follows:

(2) $$\mathrm{Sensitivity}\;=\frac{R-R_{0}}{C-C_{0}}$$

where R_0_ is the resistance value at the initial stage in air, and R is the resistance value of a certain alcohol concentration. C_0_ indicates an alcohol concentration of 0 ppm, and C represents the concentration of R. From our results, the sensitivity values of alcohol concentration from the 40 V AAO sensor at 100, 300, 500, 700 and 1000 ppm were 1.48, 1.99, 2.09, 2.45 and 2.52 Ω/ppm, respectively. The sensitivity values of AAO made of 20 V are 0.87, 1.13, 1.13, 1.24, and 1.27 Ω/ppm, which are lower than the 40 V AAO. The results indicate that as the alcohol concentration increases, the sensitivity of the AAO sensor shows a gradual increase. This suggests that at higher concentrations (1000 ppm), the detection becomes more precise. It is noted that the response values of AAO at 20 V and 40 V are 2.3% and 1.7% at 100 ppm, which is much lower than the legal detection limit of 1000 ppm. This makes a considerable contribution to environmental work safety detection or further investigations in drink driving. The linear relationship between response and alcohol concentration makes AAO alcohol sensors suitable for application in human breath testing.

In AAO alcohol sensors, there are two main factors that affect sensor performance: the nano-structure of AAO and the characteristics of the sensing substance itself. The influence of AAO nano-structure on sensor performance has been extensively discussed in our previous research [23,25]. The main conclusions are that the signal intensity of the sensor is mainly influenced by the area and thickness of the AAO. Taking resistance as an example, a smaller area and thicker AAO can result in higher signal intensity because resistance is directly proportional to thickness and inversely proportional to area. Thicker AAO also implies a larger contact area, which leads to greater signal variation and improves the sensor sensitivity. Here, AAO produced at 40V had much greater thickness, resulting in much higher resistance values. The change in resistance, represented by R-R_0_ in Formula (2), also varies significantly more due to environmental concentration changes, leading to higher sensitivity. The other structure indicator is the total circumference of AAO pores or the specific surface area, which are directly related to the sensor response and are affected by the anodization voltage. The response is a proportion of signal variation, and the higher the ratio of the contact area between the sensor pores and the test gas to the total volume, the greater the signal response [25]. At lower potentials, the pore size is smaller; however, the number of pores increases, leading to an increase in the specific surface area, which promotes an increase in the response of the sensor [25]. Therefore, the response of AAO made from 20 V is greater than 40V here.

**Figure 5:**
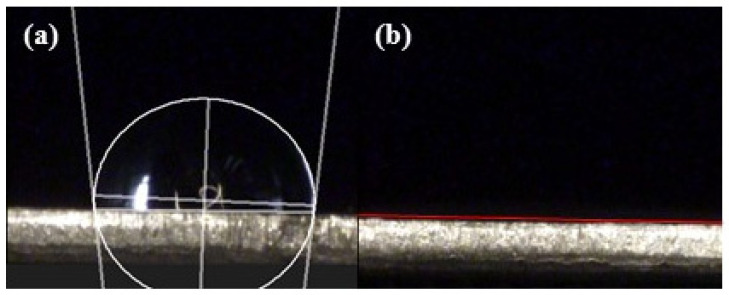
AAO sensor surface contact angle measurement: (**a**) water droplet, the contact angle is 95.2° and (**b**) alcohol droplet, the contact angle is approximately 0° with flat cover on the sensor surface.

**Figure 6:**
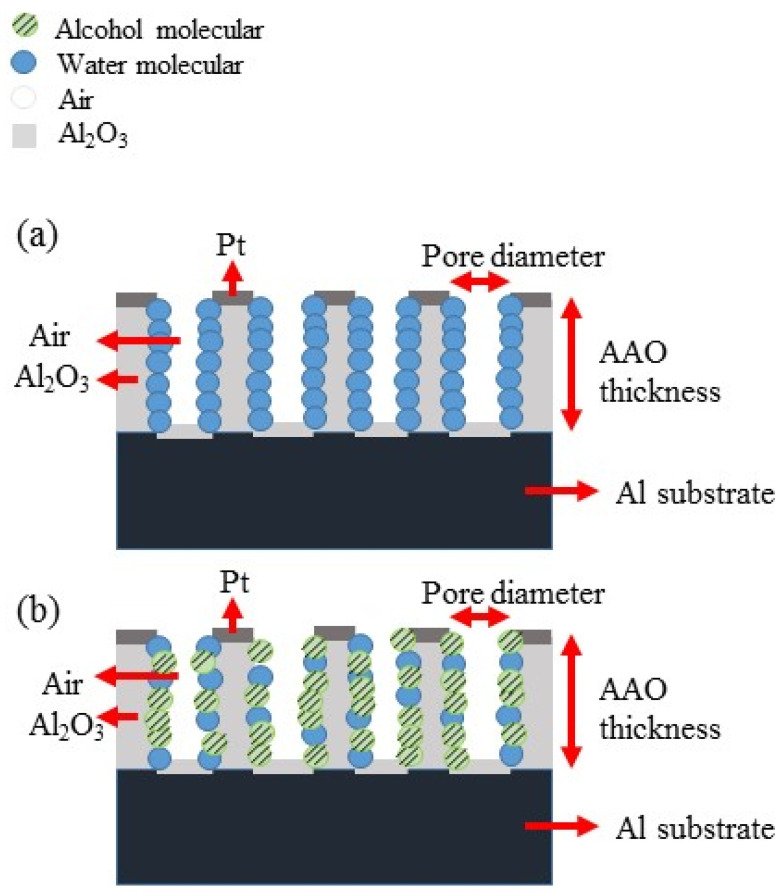
Schematic diagram of ethanol vapor molecules and water vapor molecules adsorption on the pore walls of AAO: (**a**) in air without alcohol, and (**b**) in air mixed with alcohol vapor.

The other important factor is the sensing substance itself. The alcohol not only has a higher electrical resistivity but also exhibits better adsorption on the AAO pore walls due to its superhydrophilicity compared to water, as shown in Figure 5 and Figure 6. In Figure 5, the contact angle of AAO sensor was measured. Figure 5a shows the result from water droplet and is hydrophobic at 95.2° by ImageJ software analysis, while that of alcohol is super-hydrophilic at approximately 0°, i.e., flat on the surface in Figure 5b. This indicates the alcohol vapor molecules are much more easily adsorbed on the AAO surface than the water vapor molecules. Therefore, in alcohol concentration measurements, the alcohol vapor molecules will replace the water molecules and occupy positions on the pore wall. The schematic diagram of vapor molecular adsorption is also shown in Figure 6, (a) is the AAO sensor in air, and (b) is the AAO sensor in alcohol concentration test. When more alcohol molecules replace water molecules, the resistance value will increase accordingly. This phenomenon can be explained by a simple resistance calculation Formula (3):

(3) $$R\;=\frac{\rho L}{A}$$

where ρ is the resistivity, L is the AAO thickness and A is the electrode area. In the AAO alcohol sensor measurement, L and A are constant for AAO pore structure, but ρ is a variable for the water or alcohol molecule adsorption. It is noted that the Pt may also deposit inside the pores of AAO, but it has almost no effect on the adsorption of water or alcohol molecules and the performance of the sensor. The resistivity values of DI water and alcohol molecules are 18 MΩ-cm and 19.5 MΩ-cm, respectively. When alcohol molecules replaces the water molecules, the resistance value will increase by higher resistivity of alcohol. In other words, because the resistivity and conductivity are inversely proportional, the higher resistivity of alcohol leads to poorer conductivity. As a result, at high concentrations of alcohol, the resistance of the sensor increases.

**Figure 7:**
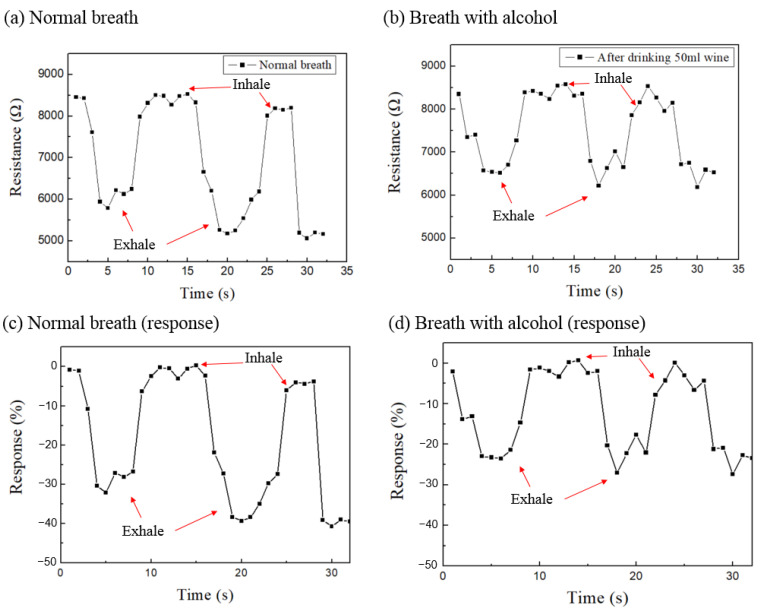
The response-time diagram of human breath measured by the AAO alcohol sensor. (**a**) Resistance value change for normal breath and (**b**) after drinking 50 mL wine (25% alcohol concentration). (**c**,**d**) The response-time diagram corresponding to (**a**) and (**b**), respectively.

Figure 7 shows the response-time diagram for human breath. Figure 7a,b show the sensing raw data from normal breath without alcohol and after drinking 50 mL wine, respectively. In normal breath in (a), the resistance the exhalation part was reduced to 5000 to 6000 Ω, however, it was only 6000 to 7000 Ω in (b) after drinking 50 mL wine. Figure 7c,d are the response-times corresponding to (a) and (b), respectively. In the inhalation part of normal breath without alcohol (Figure 7c), the response value is approximately equal to zero because the AAO sensor measures the ambient air only. However, in the exhalation part, due to the infusion of a large amount of water vapor, the response value drops rapidly to −30% to −40%. On the other hand, after drinking 50 mL wine of 25% alcohol concentration in Figure 7d, the exhaled gas contains alcohol and water vapor, and the response value is about −20% to −30%. This phenomenon can be explained by the mechanism from Figure 5 and Figure 6, where the greater resistivity and the superhydrophilicity of the alcohol molecules makes them bond more easily to the pore wall of the AAO sensor than water vapor molecules. Furthermore, the resistivity of alcohol vapor is greater than the water vapor, so the measured response changes are relatively smaller than normal breath. Measuring the gases involved in human breath is a challenging task and can be subject to various types of interference. Additionally, the amount of exhaled gas can vary from one breath to another, further complicating the measurement process. Therefore, achieving highly accurate measurements with our sensor still remain significant challenges. As shown in Figure 7a,c, it can be difficult to precisely determine the resistance value during the first exhalation, which fluctuates around 6000 ohms. However, we can still observe differences between Figure 7a,c and Figure 7b,d. Therefore, the AAO alcohol sensor we propose can measure the concentration of alcohol gas in the environment and also can be used for human breath tests, which has future application for the detection of environmental safety and drink-driving issues.

## 4. Conclusions

We successfully manufacture an AAO alcohol sensor by a simple and cost-effective one-step anodization method using commercial 1050 aluminum alloy at 20 V and 40 V under 25 °C. This process can solve the problem of complex and longer processing using other materials. AAO prepared by HPA can suppress the generation of Joule heat during reaction and shorten the process time at room temperature. The sensitivity and response of AAO resistive sensors are related to the anodizing voltage-dependent pore size and thickness. In the example of the 40 V AAO sensor for alcohol vapor detection, we find the alcohol vapor concentrations of 0, 100, 300, 500, 700 and 1000 ppm correspond to the measured resistance of 8524 Ω, 8672 Ω, 9121 Ω, 9568 Ω, 10,243 Ω, and 11,045 Ω, respectively, in a linear relationship. Furthermore, the calculated sensitivity of an AAO sensor is 1.48, 1.99, 2.09, 2.45 and 2.52 Ω/ppm at alcohol concentrations of 100, 300, 500, 700 and 1000 ppm, respectively. Sensitivity gradually increases with vapor concentration. The sensor response at a detection limit of 100 ppm is 1.7%, which is lower than the regulated value of 1000 ppm alcohol. In addition, the AAO alcohol sensor we propose applied in a human breath test to give a normal breath response of −30% to −40% and −20% to −30% after wine drinking. The resistance values reduce to 5000 to 6000 Ω in normal breath, and only 6000 to 7000 Ω in the wine drinking case. Although measuring the gases exhaled by humans poses challenges due to interferences and variations in exhaled volume, our experiments have been able to distinguish the difference between alcohol consumption and abstinence. It is worthy further application in environmental work monitoring or drink driving.

## Data Availability

Data are the coauthors’ research results and schematic drawing.
